# Combined Coronary Artery Bypass Grafting and Extra-anatomic Ascending Aorta to Bifemoral Grafting Through Median Sternotomy

**DOI:** 10.7759/cureus.6077

**Published:** 2019-11-05

**Authors:** Mohammed Al-Musawi, Michelle M Dugan, Levonti Ohanisian, David Rubay, Ali N Abed

**Affiliations:** 1 Surgery, Anschutz Medical Campus, University of Colorado, Aurora, USA; 2 Surgery, Charles E. Schmidt College of Medicine, Florida Atlantic University, Boca Raton, USA; 3 Orthopaedic Surgery, Charles E. Schmidt College of Medicine, Florida Atlantic University, Boca Raton, USA; 4 Cardiac Surgery, Iraqi Center for Heart Diseases/Medical City Teaching Complex, Baghdad, IRQ

**Keywords:** coronary artery bypass grafting, coronary artery bypass graft, bifemoral grafting

## Abstract

A high proportion of patients with severe systemic atherosclerotic disease present with the involvement of both the coronary and aortoiliac arteries. For these patients with multiple comorbidities and high surgical risk, it is critical to minimize the overall physiologic burden of the operation when possible. Furthermore, with severe or complete occlusion of vascular supply to the lower extremities, it is beneficial to avoid two-stage surgeries because of the high risk of irreversible ischemia necessitating amputation. In select cases, a single combined operation without entering the abdominal cavity may be a reliable option. We present a case with excellent results using the technique of coronary artery bypass grafting (CABG) and extra-anatomic ascending aorta to bifemoral grafting through median sternotomy and subcutaneous tunneling. Furthermore, there is a wide variation in anticoagulation reversal practices among surgeons after performing these combined grafting operations. We administered only half of the ideal calculated protamine dose for reversal of heparinization, which achieved favorable results in our patient. Overall, with symptomatic occlusion of the coronary and aortoiliac arteries, combined CABG and extra-anatomic aortobifemoral grafting with subcutaneous tunneling is a reliable surgical option. The indication for this approach should be tailored to the anatomy of the lesion and the urgency of the clinical scenario.

## Introduction

Patients with chronic cardiovascular disease frequently have systemic atherosclerotic pathology involving several anatomical regions of critical significance [1]. Notably, the prevalence of concomitant aortoiliac disease with coronary artery disease (CAD) is over 40% [2]. For these patients with multiple comorbidities and high surgical risk, the benefit of minimizing the overall physiologic burden of operative intervention is significant. As opposed to the traditional two-stage operations for coronary artery bypass grafting (CABG) and bifemoral grafting (for patients with both CAD and aortoiliac disease), the literature has shown excellent results with a combined, single-operation approach when indicated and feasible [3-7]. We present the case of a 53-year-old male who underwent CABG and aortobifemoral graft in the same operation without opening the abdominal cavity. We hope our case reinforces the option of substantially minimizing the total operative time and overall morbidity of similar patients.

## Case presentation

**Figure 1:**
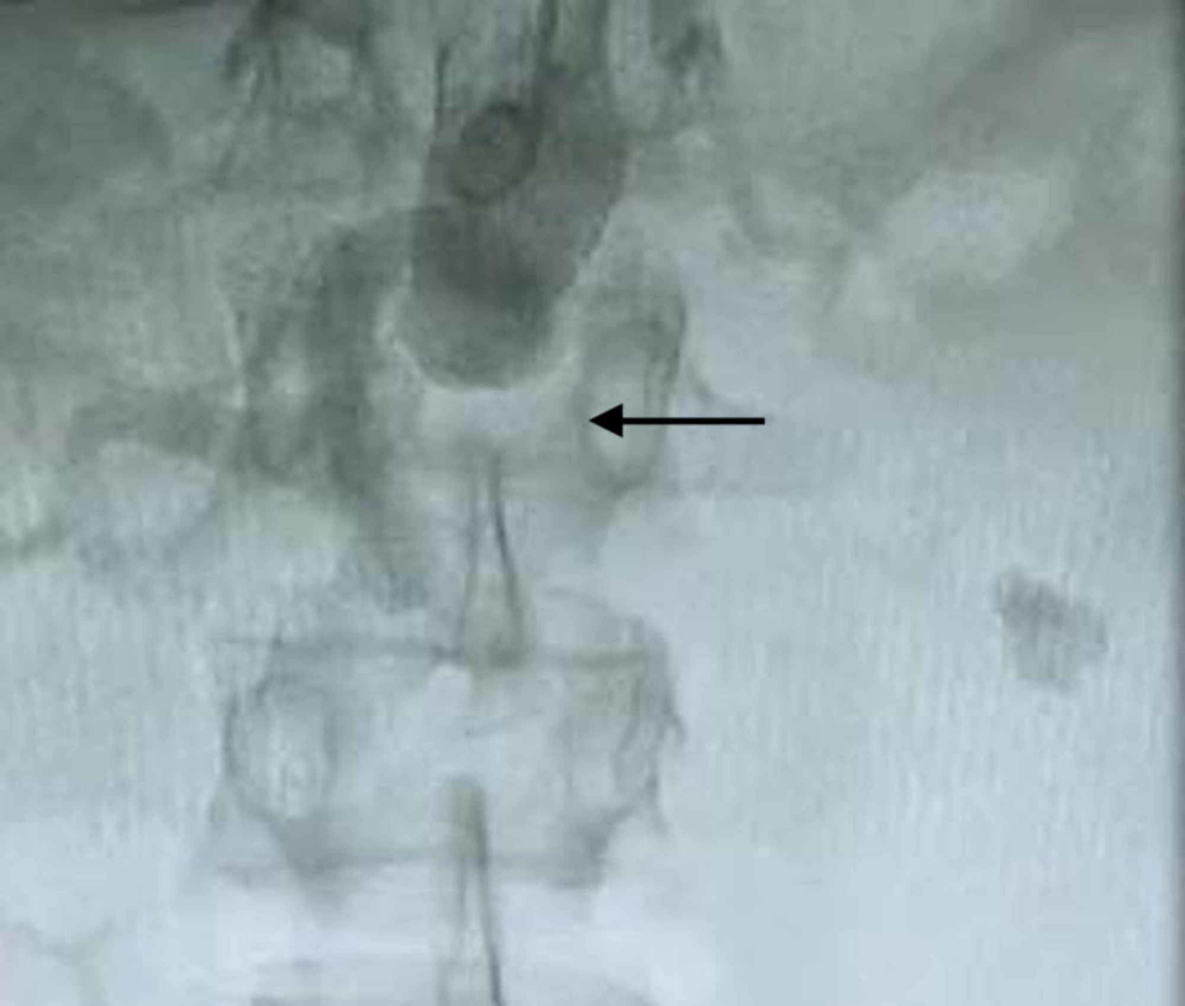
Complete occlusion with lack of flow in the abdominal aorta just inferior to the renal arteries using a radial approach.

**Figure 2:**
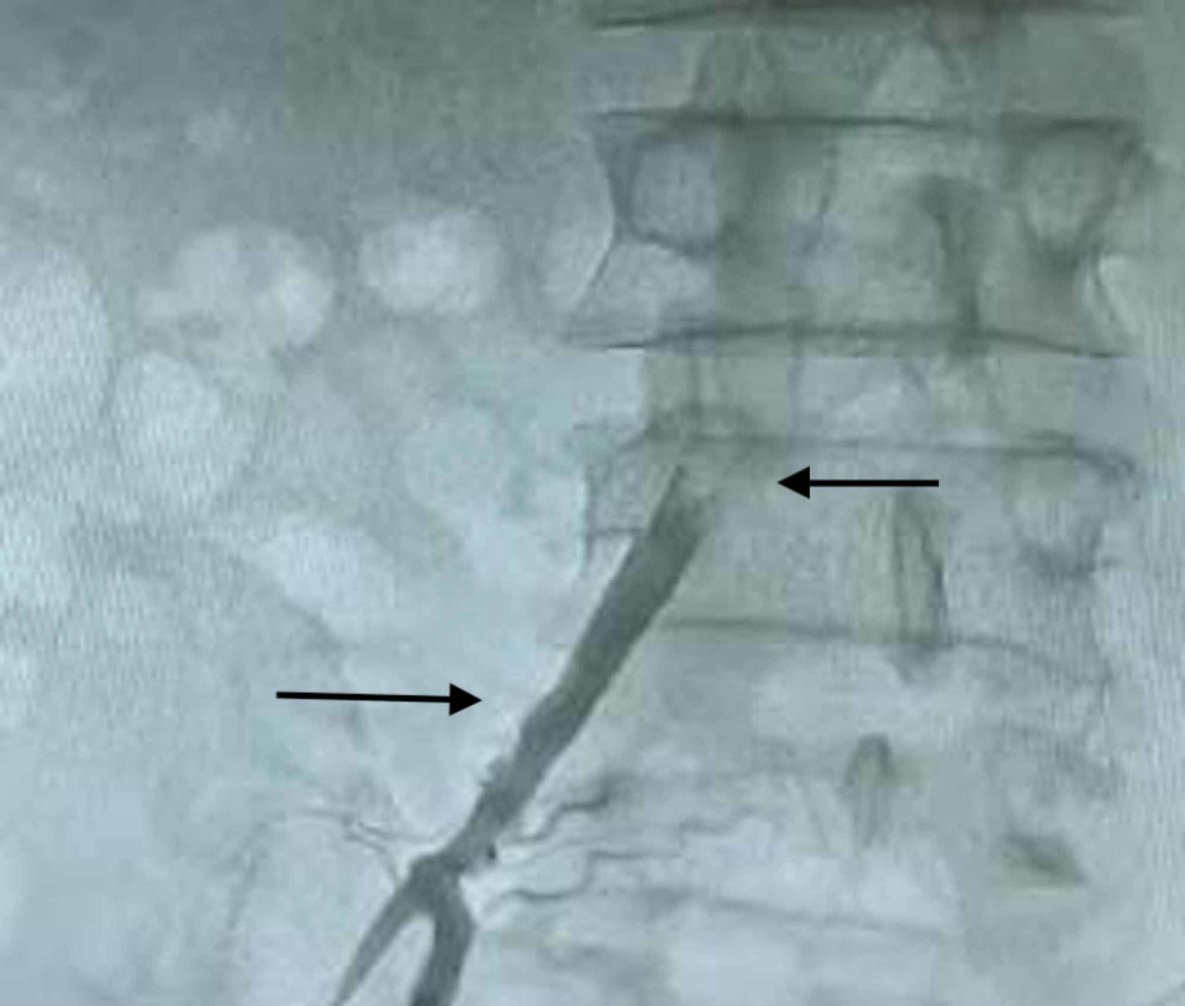
Retrograde angiography of right CIA showing patent right common iliac artery, which ends abruptly at the aortic bifurcation using a femoral approach. CIA, common iliac artery

**Figure 3:**
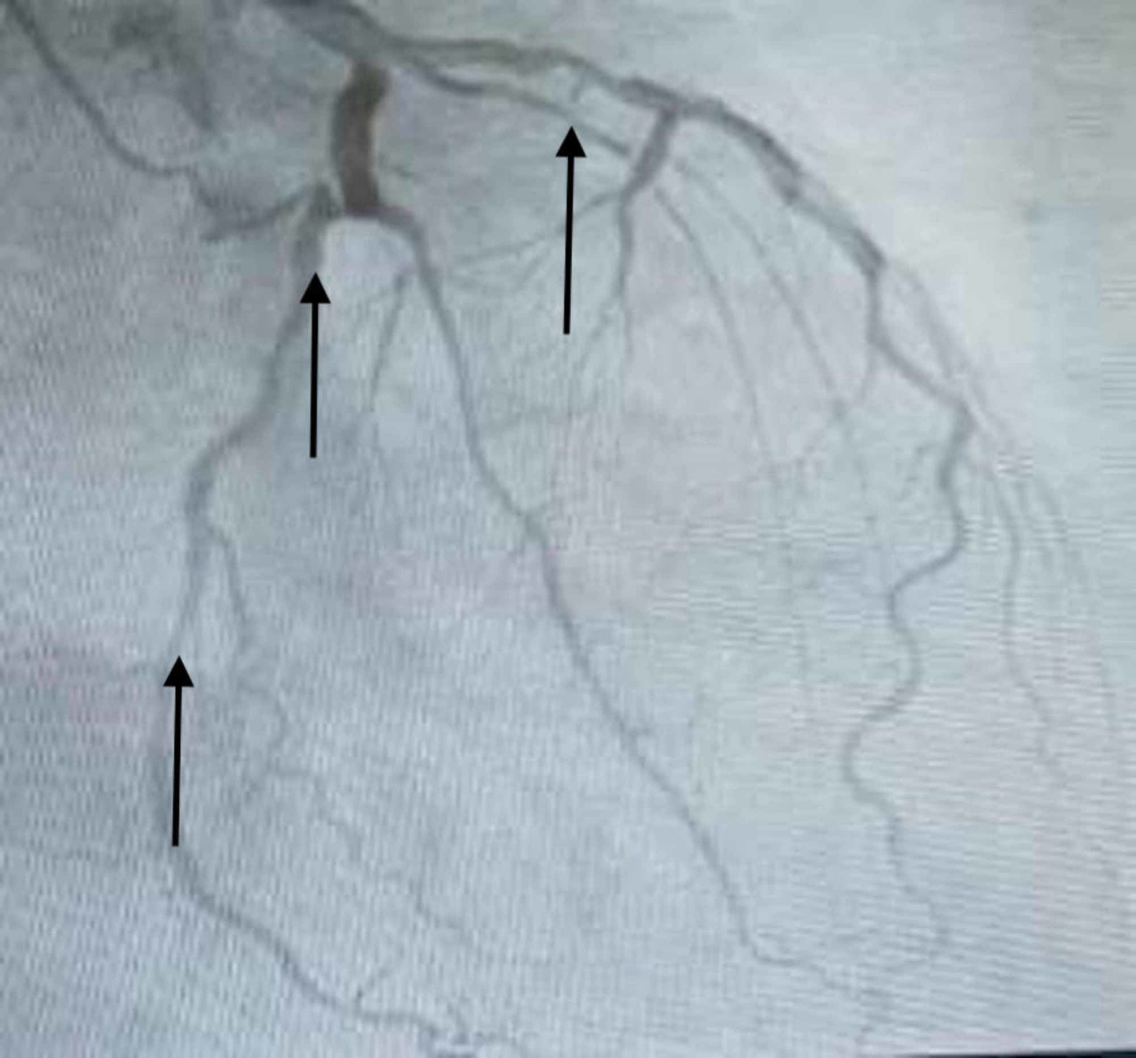
Left coronary arteries with diffuse atherosclerotic disease, showing multiple coronary artery lesions in left anterior descending and circumflex arteries.

A 53-year-old male presented with ischemic heart failure, exertional dyspnea, angina, impotence, and severe claudication with bilaterally absent femoral artery pulses. Computed tomography angiography showed three-vessel coronary disease, complete occlusion of the abdominal aorta from just distal to the renal arteries to the common iliac arteries bilaterally (Figures 1-3), and bilateral subclavian artery ostial stenosis. Given the patient’s critical condition and high surgical risk, along with well-described evidence in the literature, the decision was made to perform the CABG and aortobifemoral graft in the same operation without opening the abdominal cavity. This substantially minimized the total operative time and overall morbidity. 

**Figure 4:**
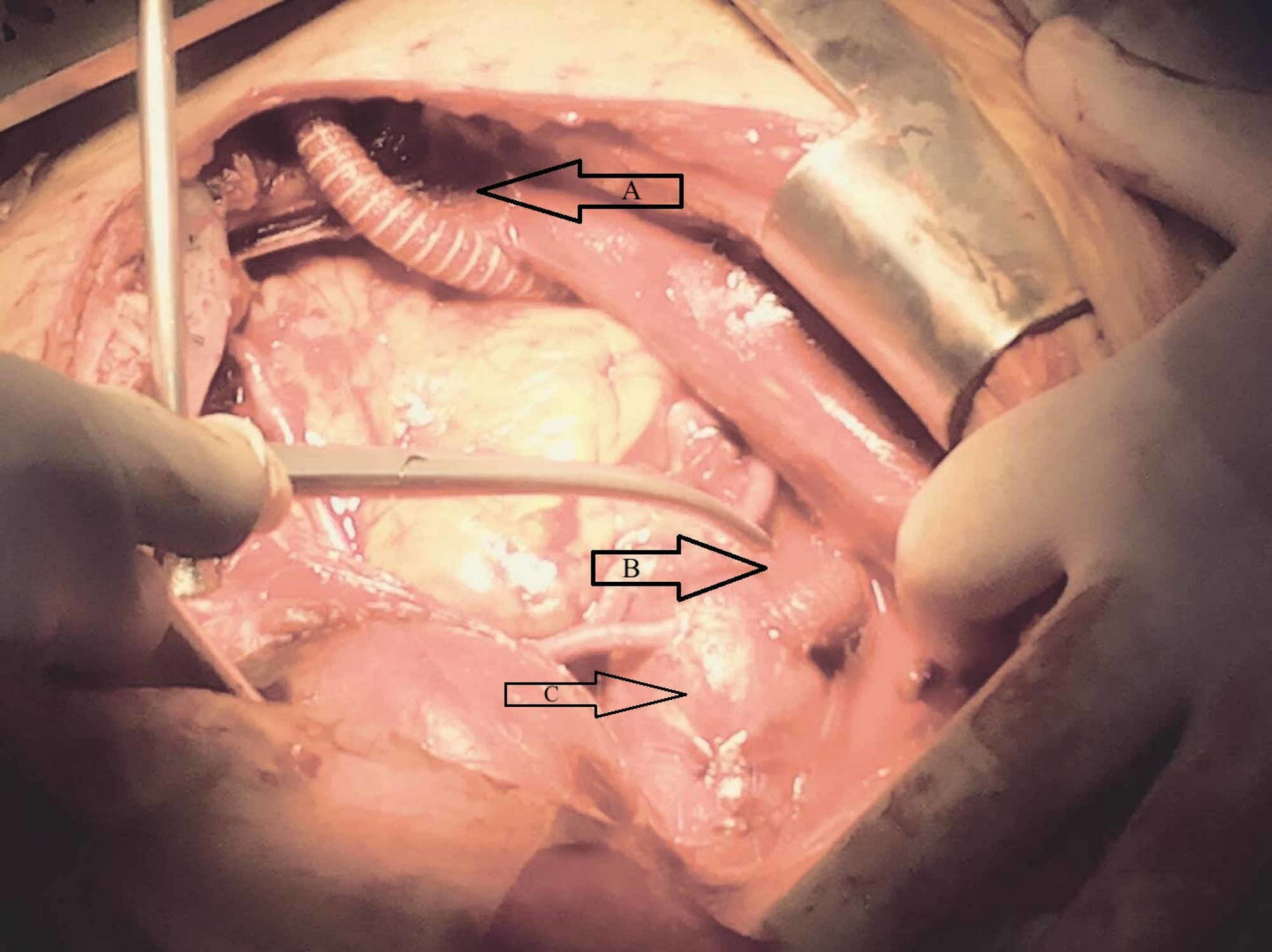
Open pericardium after completion of the proximal anastomosis of the aortobifemoral graft, showing (A) synthetic graft passed inferiorly subcutaneously in the anterior abdominal wall, (B) proximal anastomosis to the ascending aorta of the aortobifemoral graft, and (C) proximal anastomosis of the three SVG. SVG, saphenous vein graft.

The patient was placed under general anesthesia, and a conventional median sternotomy was performed. Aortocaval bypass with ascending aorta cross-clamping and cardiologic arrest were started. The grafting of each vessel was performed, connecting the saphenous vein graft (SVG) to the left anterior descending artery (the left mammary artery was not used due to left subclavian ostial stenosis), SVG to the posterior descending artery, and SVG to the obtuse marginal artery. After completing the distal anastomoses, the cross-clamp was removed, and the proximal anastomosis of the coronary graft was completed (Figure 4). 

With the CABG completed, attention was turned to the bifemoral grafting phase of the operation. Using a synthetic ringed bifurcation graft (typically used for axillobifemoral grafting), the proximal anastomosis was created on the ascending aorta. During this portion of the procedure, the aortocaval cannulas remained in place with active cardiac bypass. This provided the option of immediately placing the patient back on bypass if deterioration occurred, and also allowed us to take advantage of full heparinization during the aortobifemoral grafting. After the proximal anastomosis was completed, the synthetic graft was tunneled subcutaneously through the anterior abdominal wall and suprapubic tunnel. Right and left inguinal incisions were made to expose the femoral arteries, and the distal anastomoses of the graft were completed.

The patient was gradually weaned from bypass, and decannulation was successful. For reversal of heparinization, a half dose of protamine was used in order to improve early patency of the graft. The patient recovered well in the Cardiothoracic Intensive Care Unit and was transferred to the regular ward after 48 hours. On postoperative day 6, the patient was discharged home on dual-antiplatelet therapy. On follow-up at one, three, and six months, there was a significant improvement in the patient’s ejection fraction, angina, claudication, and impotence. 

## Discussion

Patients often present with severe systemic atherosclerotic disease with the involvement of the coronary and aortoiliac arteries [8,9]. With symptomatic occlusion of these systems, extra-anatomic bypass grafting for aortoiliac disease is an indicated and reliable surgical option [10-13]. In these select patients, the goals of preoperative planning should aim to combine interventions into one operation, avoid opening the abdominal cavity, and improve peri- and postoperative graft patency.

With severe or complete occlusion of vascular supply to the lower extremities, as in our patient, it is crucial to avoid two-stage surgeries because of the high risk of irreversible ischemia necessitating amputation of the limb(s) during the waiting period until the second operation for limb revascularization. Combining CABG with lower extremity vascular grafting (typically femoral arteries) has been well documented and supported by the literature with excellent patient outcomes [3-7]. 

It is also essential to minimize the overall physiologic burden of the operation, given the already high risk level of such cardiovascular surgeries and patient population. Opening the abdominal cavity and cross-clamping the aorta can be avoided with a proximal anastomosis outside of the abdomen and tunneling down to the lower extremities subcutaneously for the distal anastomosis [11]. This extra-anatomic bypass grafting technique minimized the technical complexity and total operation time. This strategy also helped to prevent post-laparotomy atelectasis by maintaining better ventilation and allowing for faster extubation postoperatively [13-18].

Additionally, surgical outcomes can be optimized by enhancing graft patency. In order to achieve this, we used a high inflow source and ensured aggressive anticoagulation. Evidence supports the use of aortobifemoral over axillobifemoral grafting when feasible, as both the primary and secondary patency rates are lower in axillobifemoral grafting [13-15]. The ascending aorta is already exposed in the surgical field and provides a higher flow rate [10]. Furthermore, with bilateral subclavian artery ostial stenosis, as in our patient, the axillary artery is an even less favorable graft option; it also excludes the left mammary artery as a conduit for the CABG. We utilized a unique anticoagulation strategy to further optimize graft patency. Systemic anticoagulation with activated clotting time (ACT)>300 using the cardiopulmonary bypass machine provided homogenous distribution of anticoagulation and avoided the potential ACT discrepancy above and below the arterial clamp [19]. Evidence-based data and current guidelines are lacking concerning perioperative arterial thrombosis prophylaxis with respect to these complicated cardiovascular cases, and there is a wide variation in practices among surgeons [20]. With the goal of improving early postoperative patency and minimizing risk of graft thrombosis, we used only half of the ideal calculated protamine dose to reverse anticoagulation at the completion of the operation. It was felt that the benefit of optimizing graft patency outweighed the increased risk of bleeding, and the strategy ultimately worked well in our case. 

## Conclusions

Extra-anatomic ascending aorta to bifemoral grafting through median sternotomy and subcutaneous tunneling is an effective surgical option among patients requiring combined CABG and peripheral artery revascularization. The indication should be tailored to the anatomy of the lesion and the urgency of the clinical scenario. In critical patients, the benefits of a combined single-stage operation without opening of the abdominal cavity should be carefully considered. Further research is needed to establish strong evidence-based guidelines for these complicated operations, including perioperative anticoagulation mechanisms. 
